# In vitro efficacy of N-acetylcysteine on bacteria associated with chronic suppurative otitis media

**DOI:** 10.1186/1916-0216-43-20

**Published:** 2014-07-07

**Authors:** Jane Lea, Anne Elizabeth Conlin, Inna Sekirov, Veronica Restelli, Komathi G Ayakar, LeeAnn Turnbull, Patrick Doyle, Michael Noble, Robert Rennie, William E Schreiber, Brian D Westerberg

**Affiliations:** 1 Division of Otolaryngology – Head & Neck Surgery, University of British Columbia, Vancouver, BC, Canada; 2 Department of Pathology & Laboratory Medicine, University of British Columbia, West Mall, Vancouver, BC, Canada; 3 Vancouver General Hospital, West 12th Avenue, Vancouver, BC, Canada; 4 Medical Microbiology Research Laboratory, University of Alberta Hospital, 112 Street NW, Edmonton, AB, Canada; 5 St. Paul’s Hospital Rotary Hearing Clinic, Providence 2, 1081 Burrard St, Vancouver V5Y 2B8, Canada

**Keywords:** Otitis media, suppurative, Antibacterial agents, Microbial sensitivity tests, N-acetylcysteine, Ciprodex®, Ciprofloxacin, Administration, topical, *Pseudomonas aeruginosa*, *Staphylococcus aureus*

## Abstract

**Background:**

The safety and efficacy of Ciprodex® has been demonstrated for treatment of chronic suppurative otitis media (CSOM). However, symptoms fail to resolve in 9-15% of patients. The objective of this study is to evaluate the efficacy of N-acetylcysteine (NAC) on *S. aureus,* and planktonic and sessile (biofilm forming) *P. aeruginosa* in vitro using clinical isolates from patients with CSOM.

**Methods:**

1) Stability was assessed using liquid chromatography-mass spectrometry for each component in a prepared mixture of Ciprodex® and NAC over 15 days. Sterility was assessed by measuring bacterial growth on a blood agar plate. Efficacy was assessed using a disc diffusion method by inoculating plates with *S. aureus ATCC 29513* and *P. aeruginosa ATCC 27853,* and measuring the clearance zone.

2) Fifteen *P. aeruginosa* strains were isolated from patients with CSOM and tested in vitro using the bioFILM PA™ antimicrobial susceptibility assay. Treatment solutions included Ciprodex® & ciprofloxacin +/- NAC, and NAC alone (0.25%, 0.5% & 1.25%).

**Results:**

1) NAC combined with Ciprodex® demonstrated stability, sterility, and efficacy over a two-week period

2) *P. aeruginosa* strains in the sessile (33%-40%) and planktonic (13%) state demonstrated resistance to Ciprodex® and ciprofloxacin. When NAC ≥0.5% was used in isolation or as an adjunct to either of these medications, no resistance was found in the sessile or planktonic state among all 15 strains.

**Conclusion:**

1) Ciprodex® combined with NAC has a shelf life of at least two weeks given the documented preservation of stability, sterility, and clinical efficacy of the mixed compounds.

2) *P. aeruginosa* strains demonstrated resistance to both Ciprodex® and ciprofloxacin. NAC ≥0.5% overcomes issues with resistance and shows promise in the treatment of CSOM.

## Introduction

Chronic suppurative otitis media (CSOM) is characterized by chronic inflammation of the middle ear or mastoid, a non-intact tympanic membrane, and otorrhea variably defined as lasting longer than two weeks to longer than three months [1-3]. The most common bacterial isolates in CSOM are *P. aeruginosa* (18-67% of isolates) and *S. aureus* (14-33%) [1-4].

First line pharmacologic treatment for patients with CSOM usually entails a combination antibiotic anti-inflammatory (corticosteroid) topical otic drop. The safety and efficacy of Ciprodex® (ciprofloxacin 0.3%/dexamethasone 0.1%; Bayer AG, licensed to Alcon Laboratories, Inc., Fort Worth, TX) has been demonstrated in both children and adults with CSOM [5]. However, symptoms fail to resolve in 9-15% of patients [5]. The treatment of refractory otorrhea remains a clinical challenge for otolaryngologists.

*P. aeruginosa* resistance to quinolones has been documented to be as high as 18% in suppurative otitis [6] and this coupled with recent evidence of *P. aeruginosa* biofilm formation in otology [7,8] could explain many treatment failures. Biofilm formation may sustain the inflammatory response that promotes persistent drainage from the ear [9,10].

N-acetylcysteine (NAC) is best known as an antidote to reduce liver toxicity following acetaminophen overdose [11]. However, research in the field of otolaryngology with this agent is not new. Prior studies have explored the utility of intra-tympanic NAC in the prevention of Cisplatin ototoxicity, and noise-induced hearing loss [12-15]. NAC also exhibits important mucolytic and antibacterial properties, including inhibition of *P. aeruginosa* biofilm formation [16]. A recent case series of patients with refractory otorrhea showed promising clinical results utilizing topical 2% NAC as an adjunct to Ciprodex® [17].

We deemed the combination of NAC and ciprofloxacin warranted further study. The objectives of the current study were to:

1. Delineate the in vitro stability, sterility and efficacy of a mixture of ciprofloxacin and dexamethasone (Ciprodex®) with added NAC over a two-week period to ensure safety and preservation of bacterial efficacy against *P. aeruginosa* and *S. aureus*; and

2. Assess the efficacy of NAC in combination with Ciprodex® and ciprofloxacin, and NAC alone, on *P. aeruginosa* clinical isolates from patients with CSOM with a specific focus on biofilm susceptibility in vitro.

## Materials and methods

Institutional Clinical Ethics Board approval was obtained (University of British Columbia-Providence Health Care Research Ethics Board approval number H09-00953).

### Stability, sterility and efficacy of ciprodex® in combination with 1.25% NAC

#### Study solution

A 1.25% NAC solution (12.5 mg/mL) was created by adding 50 μL of a commercially available 20% NAC solution to 750 μL of Ciprodex® (ciprofloxacin 0.3%/dexamethasone 0.1%). This solution was prepared on day 0, stored in a refrigerator at 4°C and used throughout the study to measure stability, sterility and efficacy. A 15 day study period was chosen as this corresponds to the upper limit of a typical treatment course with topical antibiotic drops for CSOM, and is the timeframe for which a standard Ciprodex® bottle will be depleted if used as directed.

#### Stability

The concentrations of ciprofloxacin, dexamethasone (in Ciprodex®) and NAC in the 1.25% NAC solution were measured on days 0, 1, 2, 7 and 15 by liquid chromatography-mass spectrometry (LC-MS).

#### Sterility data

One drop (10 uL) of the above solution was placed on a blood agar plate on days 0, 7, and 14. The plates were incubated at 35°C for 48 hours, at which time they were assessed visually for bacterial growth.

#### Efficacy data

Using a disc diffusion method, 10 μL of the 1.25% NAC and Ciprodex® mixture was inoculated onto plain discs and tested on plates containing *S. aureus ATCC 29513* or *P. aeruginosa ATCC 27853.* The same 1.25% NAC Ciprodex® mixture was used throughout this portion of the study, with separate testing occurring on day 0, 7, and 14. The plates were incubated for 24 hours at which time they were assessed for efficacy by measuring and photo-documenting the clearance zone around each disc.

### Susceptibility of P. aeruginosa isolates in the planktonic and sessile state

#### Inoculum preparation

Fifteen strains of *P. aeruginosa* isolated from clinical specimens of patients with CSOM were tested. *P. aeruginosa* ATCC 35032 was used as the quality control organism.

#### Treatment solutions

Twenty seven study solutions were prepared under sterile conditions consisting of ciprofloxacin or Ciprodex® alone (3, 1, and 0.5 μg/mL) and in combination with NAC at 0.25% (2.5 mg/mL), 0.5% (5 mg/mL) and 1.25% (12.5 mg/mL). NAC alone (no added ciprofloxacin or Ciprodex®) was also prepared at concentrations of 0.25%, 0.5% and 1.25%. Each solution was tested in duplicate. Concentrations of ciprofloxacin were chosen based on Clinical and Laboratory Standards Institute (CLSI) guidelines [18].

#### Biofilm formation

For each tested strain, a 0.5 McFarland bacterial suspension was prepared from a fresh subculture grown on blood agar plates (BAP); 300 μL of the suspension was further diluted with 19.7 mL of trypticase soy broth (TSB). The diluted suspension was used to inoculate a 96 well microtiter plate with a 95 peg inoculation lid (bioFILM PA™ antimicrobial susceptibility device; Innovotech, Edmonton, AB). One column was inoculated with sterile TSB alone for sterility control. The microplates were incubated at 35 ± 1°C on a platform shaker set at 110 rpm for 5 hours to allow biofilms to form on all pegs at a standard concentration of bacterial cells. bioFILM PA™ has received regulatory approval by Health Canada and meets all current standards of the CLSI for reproducibility and consistency. Standard protocols for testing were followed.

#### Antimicrobial susceptibility testing of *P. aeruginosa* strains

Isolates and control strain response to treatment solutions was assessed by quantifying the optical density as a gauge of bacterial growth. Optical density (OD) values of ≥0.1 using a microreader at 620 nm were considered as growth or resistance. As each solution was tested in duplicate incongruent OD values were possible; these incongruent data were excluded from the analysis and categorized as non-reproducible duplicates.

**a) Planktonic state:** After removing the microplates from the incubator, the pegged lids were carefully lifted and transferred onto the “challenge plate” containing the treatment solutions outlined above. Each concentration and combination was tested in duplicate. Immediately after transferring the pegged lid to the challenge plate, an inoculum check was performed from the growth-control wells to ensure an acceptable bacterial load of colony forming units (CFU) with a minimum goal of 5×10^5^CFU/mL. The plates were then incubated at 35 ± 1°C for 18-24 hours. Bacterial density (growth) of the planktonic population was assessed after incubation using measured optical density with a microreader at 620 nm.

**b) Biofilm (sessile) state:** After removing the challenge plates with the pegged lid from the incubator, the pegged lids were transferred to a 96-well microplate containing sterile water for 30 seconds to remove the treatment solutions from the pegs. The pegged lid was then transferred on to a 96-well microplate containing cation-adjusted Mueller-Hinton broth (CAMHB) and incubated at 35 ± 1°C for 18-24 hours to recover sessile bacteria. Bacterial density was similarly assessed after incubation using measured optical density with a microreader at 620 nm.

## Results

### Stability, sterility and efficacy of ciprodex® in combination with 1.25% NAC

#### Stability

Each component of the mixture of Ciprodex® and NAC demonstrated stability over a 15 day period. NAC concentrations varied from 1.12-1.25%, ciprofloxacin from 0.27%-0.321%, and dexamethasone from 0.0922%-0.0976% (Table 1).

**Table 1:** Stability data of N-acetylcysteine, ciprofloxacin and dexamethasone (Ciprodex combined with 1.25% N-acetylcysteine)

**Day**	**N-Acetylcysteine (%)**	**Ciprofloxacin (%)**	**Dexamethasone (%)**
**0**	1.12	0.27	0.0953
**1**	1.25	0.259	0.0976
**2**	1.2	0.258	0.0976
**7**	1.16	0.315	0.0922
**15**	1.19	0.321	0.0953

#### Sterility

The solutions remained sterile with no evidence of bacterial growth throughout the 15-day study period.

#### Efficacy

Antimicrobial effect against both *S. aureus ATCC 29513* and *P. aeruginosa ATCC 27853* remained stable based on a constant zone of inhibition around the disk containing the solution of Ciprodex and 1.25% NAC on days 0, 7 and 14.

### Susceptibility of P. aeruginosa isolates in the planktonic and sessile state

All strains grew in the control wells. One strain was slow to grow with final OD levels four times lower versus other strains, however growth was still substantial enough to demonstrate resistance to treatment with Ciprodex and ciprofloxacin (OD > 0.1), and therefore we included this strain in the analysis. Inoculum control for the challenge plates was between 6 × 10^4 and 1.7 × 10^6 cfu/mL (target: 5× 10^5 cfu/mL). Five percent of our data (42/840) was excluded from analysis as OD values were non-reproducible. The majority of the 42 non-reproducible duplicates occurred in 0.25% NAC group; the 0.25% NAC solution accounted for 23 of the 42 non-reproducible duplicates, primarily an issue in the planktonic population.

**Figure 1:**
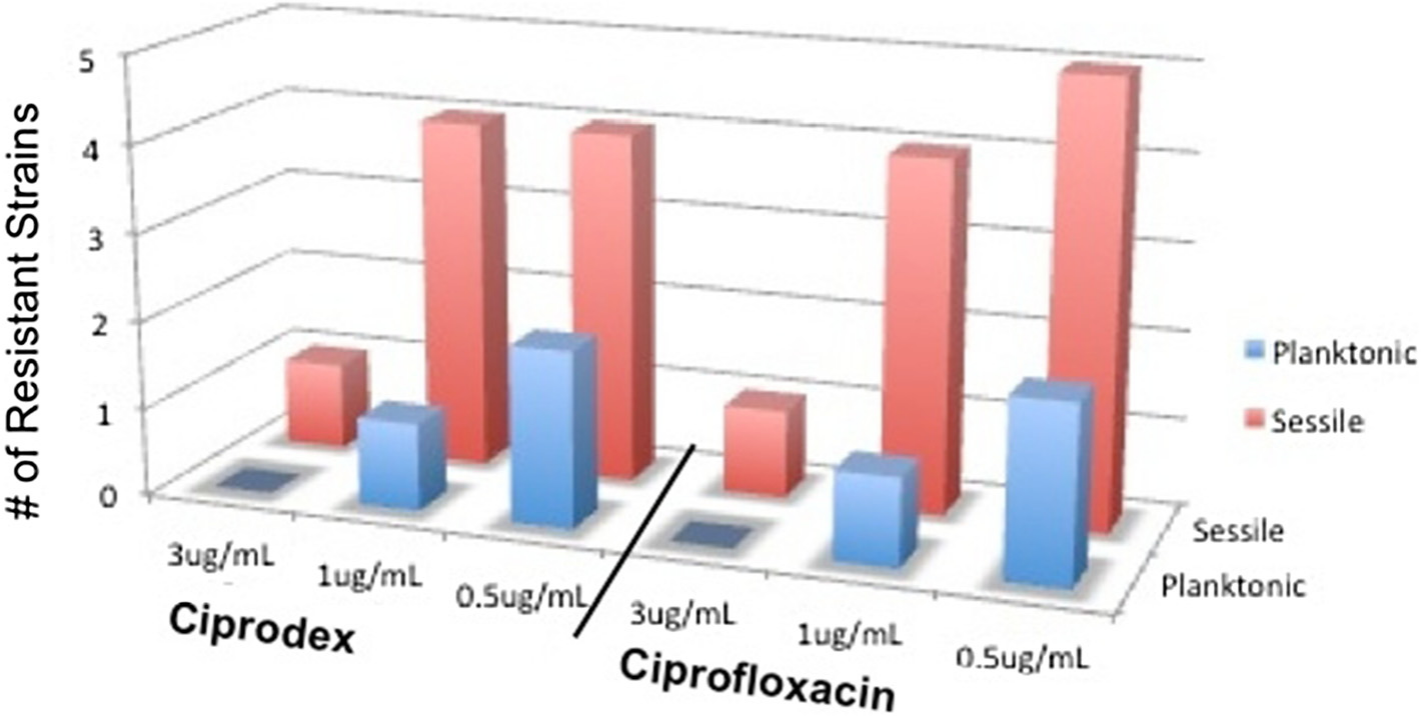
***P. aeruginosa *** **resistant strains to Ciprodex or ciprofloxacin at various concentrations.**

**Figure 2:**
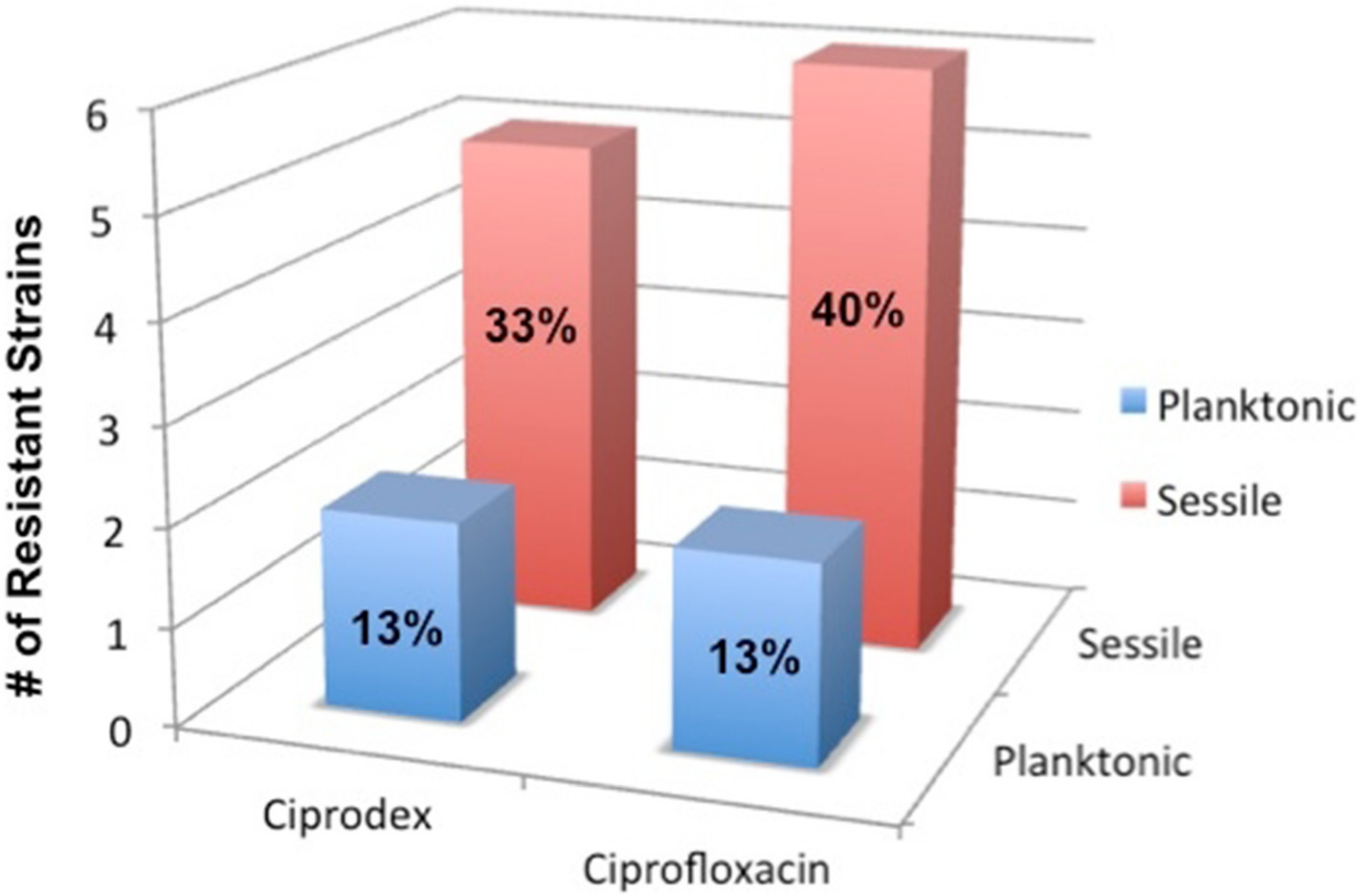
***P. aeruginosa *** **resistant strains: combined results for Ciprodex and ciprofloxacin.**

Detailed resistance rates for each concentration of Ciprodex® and ciprofloxacin are shown in Figure 1, with combined results highlighted in Figure 2. Among the 15 *P. aeruginosa* strains, two showed growth when treated with Ciprodex® or ciprofloxacin in the planktonic state, corresponding to a resistance rate of 13%. Greater resistance was seen when the bacteria were in the sessile (biofilm) state; five (33%) and six (40%) strains showed growth with Ciprodex® and ciprofloxacin, respectively (Figure 2).

**Figure 3:**
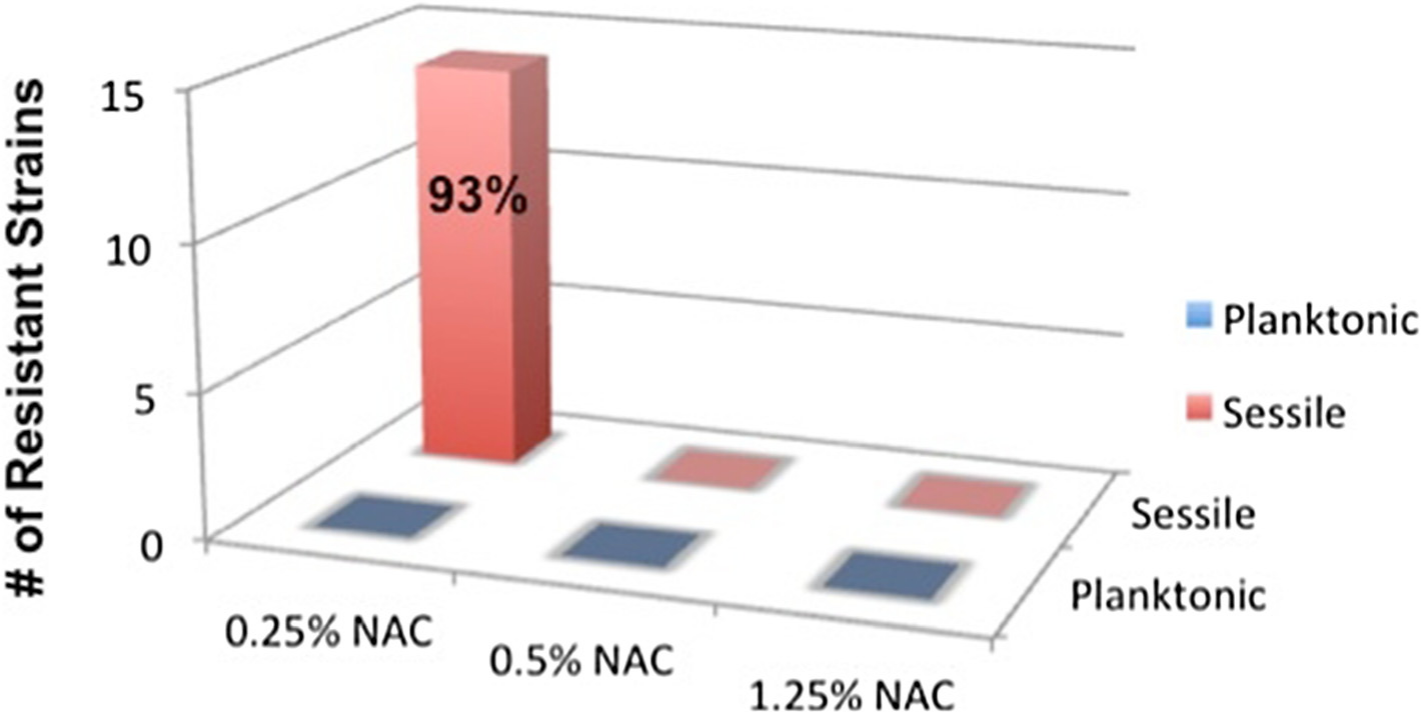
***P. aeruginosa *resistant strains to NAC alone.** Figure abbreviation: NAC: N-acetylcysteine.

All 15 planktonic and sessile *P. aeruginosa* strains were inhibited when NAC ≥0.5% was used alone (Figure 3) or as an adjunct to either Ciprodex or ciprofloxacin. Interestingly however, NAC 0.25% alone, or in combination with Ciprodex® or ciprofloxacin yielded inferior results in the sessile population. Growth occurred in all but one *P. aeruginosa* strain when NAC 0.25% was used in isolation, yielding a resistance rate of 93% (Figure 3). Thirteen strains demonstrated growth when NAC 0.25% was used in combination with either Ciprodex® or ciprofloxacin for a resistance rate of 87%. NAC 0.25% alone and in combination with either Ciprodex® or ciprofloxacin effectively inhibited growth in the planktonic population.

## Discussion

N-acetylcysteine holds promise as an adjunct in the management of patients with chronic otitis media and persistent otorrhea that is refractory to treatment with standard topical antibiotic preparations. The addition of NAC to Ciprodex® maintained a combination that was stable, sterile and efficacious in vitro against *P. aeruginosa* and *S. aureus*, up to two weeks after mixing. Furthermore, in vitro testing indicated improved efficacy against biofilm forming P. *aeruginosa* when NAC ≥ 0.5% was used alone or in combination with Ciprodex® or ciprofloxacin, overcoming the resistance observed to solutions containing ciprofloxacin alone.

This study highlights the issue of in vitro antibiotic resistance of *P. aeruginosa* to Ciprodex® and ciprofloxacin otic solutions, with resistance levels as high as 13% and 40% for planktonic and sessile states respectively. Bacteria in biofilm (sessile) states exhibit exopolysaccharide-cellular towers separated by open channels that deliver nutrients and remove waste, a complex process requiring cellular communication and modification of gene regulation, but one that renders bacteria 10-1000 times more resistant to antibiotic therapy versus genetically identical planktonic forms [19]. However, NAC at concentrations ≥0.5% revealed universal susceptibility of all *P. aeruginosa* strains from clinical isolates of patients with CSOM in both planktonic and sessile states. Our findings are consistent with other in vitro studies showing that NAC significantly inhibits the formation of bacterial biofilms when used alone. However, we failed to duplicate the beneficial synergistic effect of NAC with antimicrobial drugs that has previously been documented in the literature [16,20,21]. If the prior treatment failure rates of 9-15% in patients with CSOM [5] are attributed in whole or in part to biofilm formation, the utilization of NAC alone or as an adjunct to ciprofloxacin/dexamethasone (Ciprodex®) may culminate in improved symptom resolution and patient benefit.

Paradoxically, 0.25% NAC alone, or as an adjunct to Ciprodex® or ciprofloxacin yielded inferior susceptibility in the sessile population when compared to either Ciprodex® or ciprofloxacin alone. NAC 0.25% appeared to maintain efficacy in the planktonic state, both alone and in combined solutions with Ciprodex® and ciprofloxacin. We hypothesize that this low concentration of NAC (0.25%) may destabilize the outer surface of the biofilms to allow some viable bacteria from the inner layers to escape and grow. However, the true explanation for this finding requires further study. The biofilm is a dynamic biological complex, which may explain, in part, issues of in-vitro reproducibility.

The identification of an agent such as NAC that eradicates biofilms has potential for a significant clinical impact in the management of patients with infectious conditions in otolaryngology-head and neck surgery. A high rate of *Pseudomonas* biofilms have been identified on tympanostomy tubes in patients with persistent otorrhea despite treatment with ciprofloxacin otic drops [8]. Jang et.al, in a case series of 12 infected tympanostomy tubes revealed ciprofloxacin resistant P.aeruginosa as the sole organism with a high rate of tympanostomy tube biofilm formation as shown by EM [8]. The topical application of 20% NAC to the middle ear in patients with tympanostomy tubes has been shown to increase tube longevity, and to decrease the need for replacement of tubes, and subsequent physician visits [22]. In a recent case series of seven patients with CSOM and refractory otorrhea, use of Ciprodex® augmented with NAC (0.5% or 2%) showed resolution of symptoms in 86% (6/7) of patients within 4 weeks [17]. Compliance issues were noted in the one subject with persistent otorrhea refractory to treatment. No adverse effects were identified in treated patients with follow-up ranging from 14-19 months. In particular, serial audiometry revealed no changes in pure tone averages or speech discrimination scores, thus supporting an absence of clinically significant ototoxicity [17].

However, despite these promising findings regarding the efficacy and safety of NAC, some prudence is warranted with respect to the safety of intratympanic administration of NAC. In our study, the low concentrations of 0.5% and 1.25% NAC were utilized due to concerns about NAC at higher concentrations [12,13]. Although NAC has been shown to have protective properties against ototoxicity related to Cisplatin [12,14], as well as protection from noise induced hearing loss [15], it has paradoxically also been implicated in causing inflammation and hearing loss.

In a recent study of 20% intratympanic NAC in a guinea pig model, negative effects were seen including increased ABR thresholds, inflammation of the external auditory canal and middle ear mucosa, and diffuse osteitis with severe disruption of the organ of Corti on electron microscopy [13]. Similar inflammatory effects were found on the external and middle ear of guinea pigs treated with 4% intratympanic NAC [12]. In a guinea pig cochlear implant model [23], although preservation of residual hearing was found in the basal turn as well as a reduction in the chronic inflammatory response associated with cochlear implantation, a transient increase in hearing thresholds and osseoneogenesis was also found in animals treated with topical 4% NAC [23]. Topical 0.6% NAC after myringotomy in a guinea pig model showed significant otorrhea in the NAC treated group associated with reduced healing of the tympanic membrane post-myringotomy; 40% had persistent TM perforations in the NAC treated group versus 100% closure of perforations in the control group [24]. Two guinea pig studies on intratympanic NAC for the prevention of Cisplatin ototoxicity have demonstrated safety at NAC concentrations ≤4% [12,14]. NAC at a concentration of 4% yields nuclear/cytoplasmic membrane and stereo-cilia preservation on electron microscopy [12], and 2% NAC leads to partial preservation of distortion product oto-acoustic emissions [14]. While an inflammatory reaction was seen in the 4% NAC group [12], none was observed with 2% NAC (personal communication with Chang, KW; unpublished data).

Human studies have shown a more promising side effect profile for intratympanic NAC compared to the animal studies. Yoo et al. reported no adverse events with 2% intratympanic NAC in patients receiving cisplatin chemotherapy [25]. Riga et al, demonstrated clinical otoprotective effects of 10% intratympanic NAC in a cohort of patients treated with cisplatin based regimens based on pure tone thresholds, with the only reported adverse effect being pain post injection lasting less than five minutes (26). However, in the same study 20% intratympanic NAC was trialed in five patients but discontinued due to transient (<2 weeks) middle ear and tympanic membrane inflammation [26]. The case series by Choe et al. also demonstrates an absence of clinically significant ototoxicity based on audiometric data in seven patients treated with intratympanic NAC ≤2% [17].

The evidence appears to trend toward increased safety with reduced concentrations of topical NAC. Based on current literature, intratympanic NAC <10% appears to have an acceptable side effect profile based on clinical human studies to date. However, long-term safety profile data is currently unknown.

## Conclusion

The combination of Ciprodex® with NAC has a shelf life of at least two weeks given the documented preservation of stability, sterility and clinical efficacy of the mixture. Resistance of *P. aeruginosa* clinical isolates to both Ciprodex and ciprofloxacin was demonstrated in this study, with sessile bacteria having a higher incidence of resistance. NAC at a concentration of ≥0.5% alone, or as an adjunct to Ciprodex® or ciprofloxacin completely inhibited the growth of both planktonic and sessile *P. aeruginosa* populations. NAC 0.25% revealed paradoxical results with increased resistance in the sessile population and requires further study. NAC shows promise as an agent in treatment of bacteria implicated in CSOM in the laboratory setting. Further study is warranted to delineate its efficacy with topical administration in the clinical setting.

## Competing interests

The authors declare that they have no competing interests.

## Authors’ contributions

JL: Conducted study literature review, contributed to study design, analysis and interpretation of study data, primary author responsible for drafting and revising of manuscript. AEC: Contributed to study design, acquisition of data, analysis and interpretation of data, drafting and revising of manuscript. IS: Contributed in data aquisition, analysis and interpretation of data for biofilm studies, drafting and revising of manuscript. VR: Carried out the susceptibility testing of P. aeruginosa strains to ciprofloxacin, Ciprodex® alone or in combination with NAC, under biofilm-forming conditions, drafting and revising of manuscript. KGA: Contributed to study design, acquisition of data, analysis and interpretation of data, drafting and revising of manuscript. LT: Contributed to study design, acquisition of data, drafting and revising of manuscript. PD: Contributed to study design, acquisition of data, analysis and interpretation of data, drafting and revising of manuscript. MN: Contributed to the collection of clinical isolates of Ps. aeruginosa as well as the design, performance and interpretation of biofilm susceptibility testing, drafting and revising manuscript. RR: Contributed to development and experimental design of biofilm assays, data interpretations, drafting and revising of manuscript. WS: Contributed to design of the stability studies, data analysis, drafting and revising of manuscript. BDW: Conceptualization of study question and study design, primary coordinator of study from conceptualization to completion, interpretation of data, drafting and revising of manuscript. All authors’ read and approved the final manuscript.
